# Oxidative Stress Markers Patients with Parotid Gland Tumors: A Pilot Study

**DOI:** 10.1155/2018/4340871

**Published:** 2018-01-30

**Authors:** Pawel Sowa, Maciej Misiolek, Bartlomiej Pasinski, Grzegorz Bartosz, Miroslaw Soszynski, Monika Adamczyk-Sowa, Izabela Sadowska-Bartosz

**Affiliations:** ^1^Department of Otorhinolaryngology and Laryngological Oncology in Zabrze, Medical University of Silesia, Curie-Skłodowskiej St. 10, 41-800 Zabrze, Poland; ^2^Department of Molecular Biophysics, Faculty of Biology and Environmental Protection, University of Lodz, Pomorska St. 141/143, 90-236 Lodz, Poland; ^3^Department of Neurology in Zabrze, Medical University of Silesia, 3-go Maja St. 13-15, 41-800 Zabrze, Poland; ^4^Department of Analytical Biochemistry, Faculty of Biology and Agriculture, University of Rzeszow, Ul. Zelwerowicza 4, 35-601 Rzeszow, Poland

## Abstract

Salivary gland tumors account for 3–6% of tumors of the head and neck. About 80% of salivary gland tumors occur in parotid glands. Oxidative stress (OS) is implicated in the origin, development, and whole-body effects of various tumors. There are no data on the occurrence of OS in the parotid gland tumors. The aim of this study was to ascertain if whole-body OS accompanies parotid gland tumors, based first of all on oxidative modifications of blood serum proteins and other markers of OS in the serum of the patients. The group studied included 17 patients with pleomorphic adenoma, 9 patients with Warthin's tumor, 8 patients with acinic cell carcinoma, and 24 age-matched controls. We found increased concentration of interleukin 4 in patients with acinic cell carcinoma, decreased plasma thiols, increased AOPP concentration, and decreased FRAP of blood serum in all groups of the patients while protein oxidative modifications assessed fluorimetrically, protein carbonyls, protein nitration, malondialdehyde concentration, and serum ABTS^⁎^-scavenging capacity were unchanged. These data indicate the occurrence of OS in patients with parotid gland tumors and point to various sensitivities of OS markers.

## 1. Introduction

For the past 45 years or so, oxidative stress (OS) has been increasingly recognized as a contributing factor in aging as well as in various forms of pathophysiology such as tumors [1].

Salivary gland tumors account for 3–6% of tumors of the head and neck. About 80% of salivary gland tumors occur in parotid glands, 10–17% of which are malignant [2]. The tumor is seen predominantly among white people, mainly between the 5th and 6th decade of life. Males suffer twice as often as females [3].

The parotid glands are the largest salivary glands in humans and are frequently involved in disease processes. Factors that increase the risk of parotid gland cancer include older age. In recent years, there has been a significant increase in the number of patients hospitalized and operated due to parotid salivary tumors. Over the past few years, in the Department of Otorhinolaryngology and Laryngological Oncology in Zabrze (Poland), we have been operating more than twice as many patients per year compared to previous years. Although some reports indicate possible use of cellular phones as an etiological factor [4–6], others deny such observations [7].

The vast majority of parotid salivary tumors are benign, with pleomorphic adenoma (PA, 60% of all benign salivary gland tumors) and Warthin's tumor (WT), also known as adenolymphoma or papillary lymphomatous cystadenoma (5–30% of all salivary gland tumors) [8]. Pleomorphic adenoma is the most common benign mixed salivary gland neoplasm. It has diverse histological presentation and occurs in both major and minor salivary glands [9]. Warthin's tumor is more common in male patients (4 : 1 male : female ratio) during the sixth and seventh decades of life [10, 11]. Unlike other benign neoplasms of the salivary glands, this tumor has a tendency towards bilateral involvement, and approximately 90% of lesions occur in the superficial lobe of the parotid gland [11, 12].

Although malignant tumors in the salivary glands represent a definite minority (about 10%), it is important to remember that some benign tumors may undergo malignant transformation (e.g., about 5% of pleomorphic adenoma) [13]. Acinic cell carcinoma (ACC) of salivary gland represents 2.5%–5% of all parotid gland neoplasms [14]. Acinic cell carcinoma occurs in the parotid gland more often than primary planoepithelial carcinoma. In comparison to other salivary gland malignancies (adenoid cystic carcinoma or salivary duct carcinoma), ACC malignancy is quite low. This is reflected in the high level of 10-year survival rates (70%) [3].

Oxidative stress is implicated in the origin, development, and whole-body effects of various tumors [15–17].

The most commonly used markers of OS include increased levels of oxidation products of lipids, proteins and nucleic acids and decreased concentrations of antioxidants. The blood serum concentration of malondialdehyde (MDA) is assayed most frequently, although this assay is often criticized due to its low specificity [18]. Among markers of protein oxidation, decreased protein thiol group content and increase of protein carbonyl content are most often determined. Since low-molecular thiols are very low in blood serum, serum thiol groups represent almost exclusively protein thiols [19]. Advanced Oxidation Protein Products (AOPP) have been proposed as another marker of protein oxidative damage [20]. We have used fluorimetric measurements to evaluate the increase in tryptophan and tyrosine oxidative degradation products, such as N-formylkynurenine, kynurenine and dityrosine, and glycophore, the product of protein glycoxidation [21, 22]. The nitrotyrosine content is a measure of protein modifications by reactive nitrogen species, mainly peroxynitrite [23]. Decreases in concentrations of individual antioxidants, for example, ascorbic acid, are sensitive indices of oxidative stress; another parameter evaluated in this respect is the total antioxidant capacity (TAC) of blood serum [24]. 

There are no data on the occurrence of OS in the parotid gland tumors. The aim of this study was to ascertain if whole-body OS accompanies parotid gland tumors, based first of all on oxidative modifications of blood serum proteins including protein thiol group level and AOPP and other markers of OS in the serum of the patients with parotid gland tumors such as total antioxidant activity (TAC) of the serum.

## 2. Materials and Methods

### 2.1. Patients with Salivary Gland Tumors and Study Design

**Figure 1:**
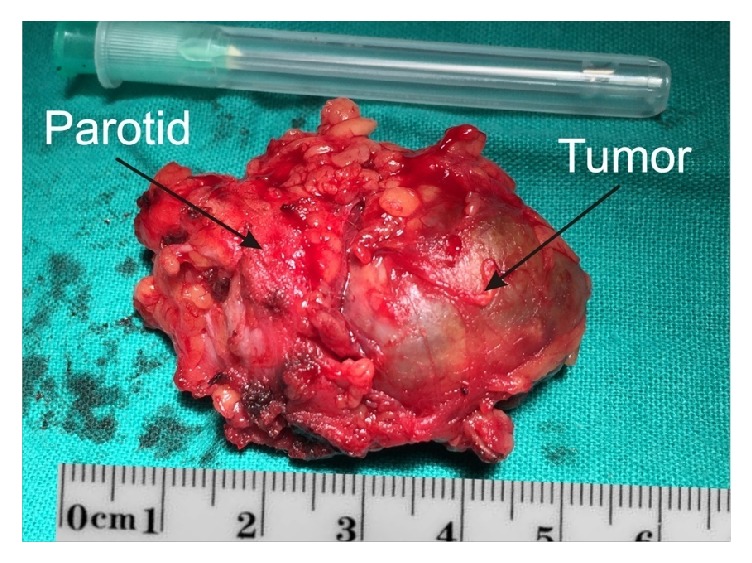
Pleomorphic adenoma of parotid gland: the intraoperative view after removing of the tumor (Tumor) together with a part of superior lobe of parotid glad (Parotid).

The experiment was planned prospectively. Among all nonsmokers patients admitted to the Department of Otorhinolaryngology and Oncological Laryngology in Zabrze, Medical University of Silesia in Katowice, Poland, we selected those suffering from parotid gland tumor at the turn of 2016 and 2017; thus we initially examined 86 individuals. Patients with previous oncological treatment, diabetes mellitus, or obesity were excluded from the study. We further examined 36 of those patients. Venous blood samples (5 ml each) were taken, centrifuged, frozen, and stored in −80°C until the final examination. Selected patients were divided into groups according to the postsurgical pathological results. Therefore we included 17 patients into pleomorphic adenoma (PA) group (Figure 1), 9 of them into Warthin's tumor (WT) group, and 8 into acinic cell carcinoma (ACC) group. Control group consisted of 24 age-matched healthy subjects. The remaining patients were excluded from experiment because of not clear pathological result or because of too small number of individuals to create a separate group.

**Figure 2:**
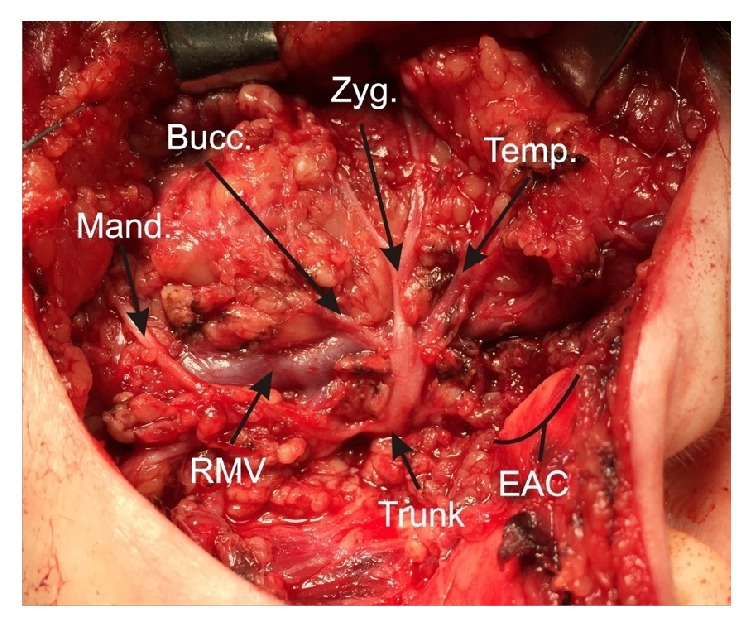
Surgical view of the left facial nerve (n. VII) after removal of the tumor and superficial lobe of the parotid gland. The main trunk (Trunk) of the nerve and its main branches: temporal (Temp.), zygomatic (Zyg.), buccal (Bucc.), and mandibular (Mand.) were marked by arrows. Cartilage of external auditory canal was marked as EAC and retromandibular vein as RMV.

The basic demographic and clinical characteristics of parotid cancer patients are summarized in Table 1. A preoperative fine-needle aspiration biopsy and ultrasonography were performed routinely in all patients. A superficial parotid lobectomy was performed when the tumor was located in the superficial lobe of the parotid gland. In cases with involvement of the deep lobe, total parotidectomy was performed. The term parotidectomy should be used when two conditions are fulfilled: dissection of the facial nerve (at least the main trunk and one the two major divisions) and removal of at least one level (Figure 2). Patients were operated by the same surgeon. Parotidectomy remains the mainstay of treatment for both benign and malignant lesions of the parotid gland. There exists a wide range of possible surgical options in parotidectomy in terms of extent of parotid tissue removed. Parotidectomies levels were graded according to the European Salivary Gland Society (ESGS) classification as follows: (I) lateral superior, (II) lateral inferior, (III) deep inferior, (IV) deep superior, and (V) accessory. The type of resection is divided into formal parotidectomy with facial nerve dissection and extracapsular dissection [25].

### 2.2. Materials

All basic reagents were from Sigma-Aldrich (Poznań, Poland), unless indicated otherwise. OxiSelect™ Protein Carbonyl Fluorometric Assay Kit (STA-310) was obtained from Cell Biolaboratories. 3-Nitrotyrosine in the proteins of human serum was determined by an ELISA method (Catalog# K7829; Immundiagnostik AG, Bensheim, Germany). Human IL-4 Quantikine ELISA Kit (Catalog# D4050) was purchased from R&D System (Minneapolis, MN, USA). Salivary *α*-amylase activity was assayed using an Amylase Activity Colorimetric Assay Kit (Catalog# K711-100; BioVision).

Fluorimetric and absorptiometric measurements were done in a Tecan Infinite 200 PRO multimode reader (Tecan Group Ltd., Männedorf, Switzerland) or in an EnVision Multilabel Plate Reader (Perkin-Elmer, Überlingen, Germany). All measurements were performed in triplicate and repeated a minimum of three times.

### 2.3. Blood Sampling

Samples of venous blood (5 ml) from tumor-bearing untreated patients (before surgery) and controls were collected into serum-separating tubes and immediately centrifuged to isolate serum. Collected serum samples were stored at −80°C until biochemical analysis, for no more than 2 months. They were thawed at room temperature only once at the time of analysis.

### 2.4. Determination of *α*-Amylase Activity

The *α*-amylase activity was determined using the Amylase Activity Colorimetric Assay Kit (BioVision) according to the instruction of the manufacturer.

### 2.5. Estimation of Interleukin 4 Concentration

Serum IL-4 concentration was estimated by an enzyme immunoassay (ELISA) using Quantikine Human IL-4 Diagnostics Kit.

### 2.6. Estimation of Protein Carbonyls and Protein Nitration

The content of protein carbonyls and protein 3-nitrotyrosine were estimated using OxiSelect™ Protein Carbonyl Fluorometric Assay Kit and 3-Nitrotyrosine ELISA kit, respectively, according to the protocols supplied by the manufacturers.

### 2.7. Estimation of Protein Oxidative Modifications

Products of oxidative modifications of proteins were estimated on the basis of their characteristic fluorescence. Fluorescence measurements were done by applying 150 *μ*l of the serum diluted 1 : 50 with phosphate-buffered saline (PBS; 1 tablet of PBS/100 ml H_2_O) on wells of a 96-well plate. Fluorescence was measured at wavelengths of 325/440 (AGEs), 330/415 (dityrosine), 325/434 (N′-formylkynurenine), 365/480 (kynurenine), and 295/340 nm (tryptophan) [21, 22].

### 2.8. Estimation of Thiol Groups

Thiol groups were estimated using a modification of the Ellman's method [26]. Samples (20 *μ*l) were pipetted to wells of a 96-well plate containing 100 *μ*l of 0.1 M phosphate buffer, pH 8.0. Afterwards, 2 *μ*l of 10 mg/ml Ellman's reagent [5,5′-dithiobis-(2-nitrobenzoic acid); DTNB] was added. Absorbance was measured after 1 h incubation in the dark at 37°C at the wavelength of 412 nm against a reagent blank. The thiol group content was calculated on the basis of a standard curve using glutathione as a standard.

### 2.9. Estimation of Protein

The protein concentration was estimated using the method of Lowry et al. [27]. Serum diluted 200 times with PBS (100 *μ*l) was mixed with 500 *μ*l of the Lowry reagent (formed by mixing 30 ml of 2% Na_2_CO_3_ in 0.1 M NaOH, 0.6 ml of 5% C_4_H_4_O_6_KNa·4H_2_O, and 0.6 ml of 2% Cu_2_SO_4_) and incubated at room temperature for 10 min. Afterwards, 50 *μ*l of the Folin–Ciocalteu reagent was added, and the plate was shaken and incubated at room temperature for 30 min. The absorbance was measured at 750 nm. Standard curve was prepared with human serum albumin (0–300 *μ*g/ml).

### 2.10. Estimation of Malondialdehyde (MDA)

Serum MDA concentration was assayed according to Ohkawa et al. [28]. Briefly, the serum samples (50 *μ*l serum plus 50 *μ*l PBS or 100 *μ*l PBS blank) were mixed with 200 *μ*l of ice-cold mixture of 0.37% thiobarbituric acid (TBA) and 15% trichloroacetic acid (TCA) in HCl to precipitate protein. The reaction was performed at pH 2-3 at 100°C for 40 min. The precipitate was pelleted by centrifugation at 3000 ×g at 4°C for 10 min. Absorbance of the supernatants was read at a wavelength of 532 nm.

The majority of TBA-reactive substances (TBARS) is MDA; thus the concentration of MDA in blood plasma was expressed in *μ*M. The results were calculated using an absorption coefficient for MDA of 1.56 × 10^5^ M^-1 ^cm^−1^

### 2.11. Estimation of AOPP

Advanced oxidation protein products (AOPP) were estimated using the method of Witko-Sarsat et al. [20]. Briefly, 200 *μ*l of serum diluted 1 : 5 with PBS was applied to the 96-well plate and 20 *μ*l of acetic acid was added to each well. Absorbance was measured at 340 nm against a blank containing 200 *μ*l of PBS, 20 *μ*l of acetic acid, and 10 *μ*l of 1.16 M potassium iodide. Calibration curve was prepared using chloramine T at concentrations of 0–100 *μ*M by applying 200 *μ*l chloramine T, 20 *μ*l acetic acid, and 10 *μ*l of 1.16 M potassium iodide to the plate. AOPP concentration is expressed in nmol chloramine T-equivalents/mg protein.

### 2.12. Estimation of Total Antioxidant Capacity of Blood Serum as FRAP

The serum a total antioxidant status was measured in serum using the ferric reducing antioxidant power assay (FRAP). The Ferric Reducing Antioxidant Potential assay measures the ability of antioxidants to reduce ferric (Fe^3+^) ions to ferrous (Fe^2+^) ions [29]. 0.3 M acetate buffer (pH = 3.6), 0.01 M TPTZ (2,4,6-tripyridyl-s-triazine) in 0.04 M HCl, and 0.02 M FeCl_3_*∗* 6 H_2_O mixed in 10 : 1 : 1 and 180 *μ*l of this mixture were added on wells of a 96-well plate containing 10 *μ*l of sample and 10 *μ*l of PBS. The reduction of Fe^3+^-2,4,6-tripyridyl-s-triazine complex to the ferrous form at low pH was monitored by measuring the absorption change after 20 min incubation in room temperature at 593 nm. The value was calculated relevant to the activity of Trolox and expressed as *μ*moles Trolox equivalents/l (*μ*M).

### 2.13. Estimation of Total Antioxidant Capacity with ABTS^*∗*^

Antiradical activity is a measure of the ability of a given compound to react with free radicals. One stable free radical employed in such reactions is the 2,2′-azinobis (3-ethylbenzothiazoline-6-sulfonic acid) radical  (ABTS^*∗*^). Standard antioxidants react rapidly with ABTS^*∗*^ (within seconds; “fast antioxidants”) while some react at a lower rate (“slow antioxidants”) [24]. Briefly, 2 *μ*l of sample and 18 *μ*l of PBS were added to a solution of ABTS^*∗*^, diluted, such that 200 *μ*l of the solution had absorbance of 1.0 in a microplate well. The decrease in ABTS^*∗*^ absorbance was measured after 1 min (“fast” scavenging) and between 10 and 30 min (“slow” scavenging) of incubation at ambient temperature (21 ± 1°C) at 414 nm. FRAP value was calculated relevant to the activity of Trolox and expressed as *μ*moles Trolox equivalents/l (*μ*M).

### 2.14. Statistical Analysis

All experiments were performed at least in triplicate. Data are shown in the form of arithmetic mean values and standard deviations. Differences between means were analyzed using Kruskal–Wallis test. The statistical analysis of the data was performed using STATISTICA, version 12.5, (StatSoft, Inc., http://www.statsoft.com).

## 3. Results and Discussion

Serum amylase activity, expected to be altered in the patients, did not show consistent changes in patients with parotid gland tumors. There was a tendency for decrease in the WT and, especially, in the PA groups but they did not reach the level of statistical significance due to high interindividual variability (not shown).

**Figure 3:**
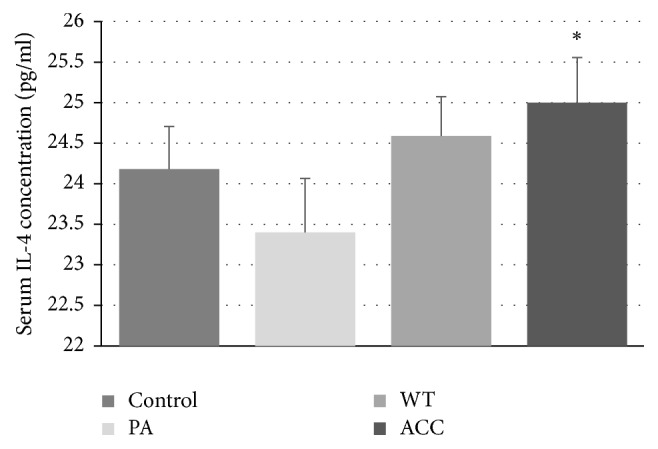
Serum IL-4 concentration in patients with parotid gland tumors; ^*∗*^*P* < 0.05.

Serum IL-4 concentration changed differently in different groups of parotid tumor patients, having a tendency for decrease in PA patients (not reaching statistical significance), not changing in WT patients and increasing in ACC patients (Figure 3). As IL-4 is involved in the stimulation of activated B-cell and T-cell proliferation and the differentiation of B cells into plasma cells [30], the increase of its level in ACC patients points to changes in the immune system induced by the carcinoma. Il-4 has been demonstrated to suppress the development of tumor development in an atopic melanoma model [31] so the observed elevation of IL-4 level in ACC may reflect the attempt of the organism to combat the tumor.

**Figure 4:**
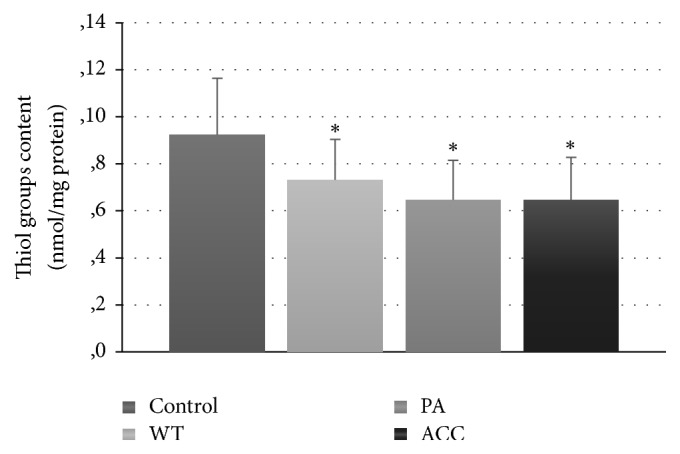
Serum thiol group content in patients with parotid gland tumors; ^*∗*^*P* < 0.05.

**Figure 5:**
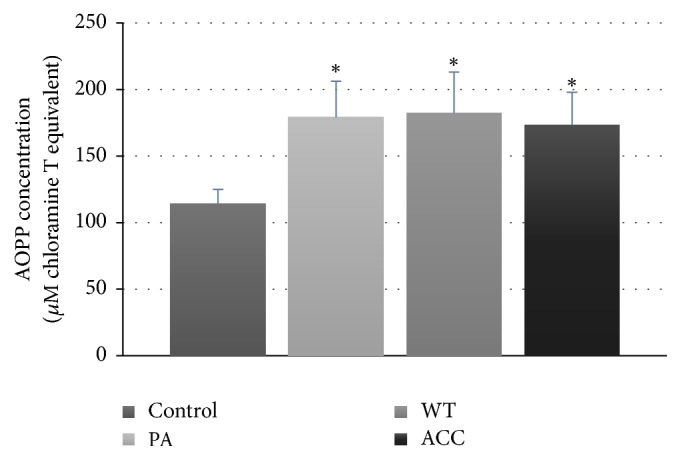
Serum AOPP concentration in patients with parotid gland tumors; ^*∗*^*P* < 0.05.

Fluorimetric parameters of protein glycoxidation (tryptophan fluorescence, the levels of glycophore, dityrosine, N-formylkynurenine, and kynurenine) were not significantly altered with respect to the control groups (not shown). However, the level of thiol groups decreased (Figure 4) and the AOPP level was elevated (Figure 5) in all groups of parotid tumor patients.

Most of total blood plasma proteins are protein thiols (400–600 *μ*M), predominantly Cys34 of serum albumin, which is reduced in about 75%, while low-molecular weight thiols constitute only 12–20 *μ*M. Plasma protein thiols react nonenzymatically with various reactive oxygen species so their level is decreased under OS conditions [19].

AOPPs are the dityrosine-containing and crosslinking protein products formed during OS by reaction of plasma protein with chlorinated oxidants and often carried by albumin* in vivo*. Accumulation of plasma and renal AOPPs is a common pathologic finding in chronic kidney disease patients but is also implicated in the pathogenesis of atherosclerosis and cardiovascular events [32]. The present results demonstrate that they may be a useful marker of OS also in other diseases.

The level of protein carbonyls and protein nitrotyrosine was not significantly altered with respect to the control group. Protein carbonyls had a tendency to increase in the PA group (not shown) while the nitrotyrosine level had a tendency to decrease in the PA and WT groups (not shown) but these changes were devoid of statistical significance. Interestingly, when data for males and females were evaluated independently, statistically significant differences were noted in the level of carbonyls in the WT group (females versus males: 0.73 ± 0.16 versus 0.47 ± 0.07 arbitrary units (au), *P* < 0.05), in the PA group (0.76 ± 0.14 versus 0.53 ± 0.22 au, *P* < 0.05), and in the ACC group in the level of nitrotyrosine (3.67 ± 0.78 versus 1.89 ± 1.11 au). There were no significant differences in any parameters between the controls. In the case of the PA group, the carbonyl level was significantly elevated (*P* < 0.05) with respect to the control (0.76 ± 0.14 versus 0.66 ± 0.19 au, *P* < 0.05). These data suggest that the level of OS is higher in females than in males in parotid cancer patients.

These data demonstrate whole-body oxidative stress patients with all parotid gland tumor patients studied but various parameters of oxidative protein modifications are not equally sensitive to detect it, the most sensitive being the protein thiol level and AOPP.

**Figure 6:**
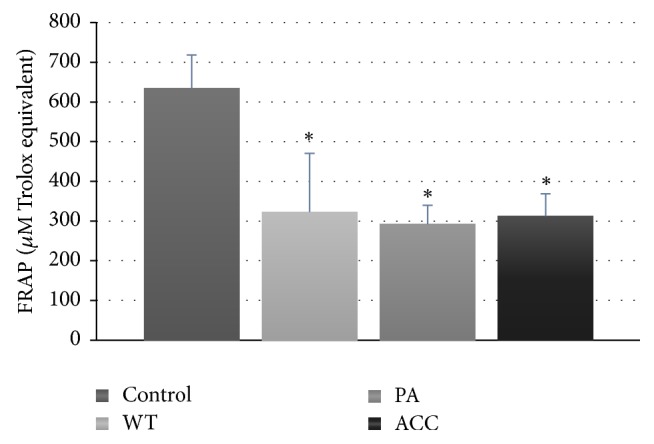
Serum FRAP in patients with parotid gland tumors; ^*∗*^*P* < 0.05.

Other oxidative stress markers studied included serum MDA concentration and total antioxidant capacity (TAC) of the serum measured as TRAP and ABTS^*∗*^ scavenging capacity. Although MDA concentration had a tendency to increase in the PA and WT groups and a tendency to decrease in the ACC groups, the differences measured did not reach statistical significance (not shown). Both “fast” and “slow” ABTS^*∗*^ scavenging capacity of blood serum of the patients were unchanged with respect to control (not shown). However, FRAP was decreased in all groups of patients studied (Figure 6). The difference between the results of TAC of blood serum by ABTS^*∗*^ scavenging and FRAP methods is striking. However, it should be taken into account that antioxidants present in serum differently contribute to results of TAC measurements by both methods. Uric acid contributes in 61.7% to the FRAP assay and only in 19.3% to results of ABTS-based assays, and the contribution of tocopherol is 5.8 and 1.7%, respectively, that of ascorbic acid 10.1 and 3.1%, respectively, and that of bilirubin 4.3 and 1.0%, respectively [33]. It is still unclear why decrease in protein thiols is not reflected in decreased ABTS^*∗*^ scavenging capacity of blood serum of the patients, but albumin (mainly albumin thiols) contributes in only 28% to the ABTS^*∗*^ scavenging capacity of blood serum while other antioxidants are responsible for 46.9% of this capacity and increase in the level(s) of some of them might have contributed to the result obtained. What concerns FRAP, the decrease in protein thiols had to contribute to its decreased value but, since FRAP is dependent on albumin only in 7.3% [33], decreased concentrations of other antioxidants had to have its share in decreased FRAP values in the patients. In any case, FRAP, previously documented to be decreased in many diseases involving oxidative stress [24], is sensitive enough to detect whole-body oxidative stress in patients with parotid gland tumors.

While the level of IL-4 was different on various patient groups, alterations in parameters reflecting OS were similar in all groups, which suggests that the level of OS is the same in all groups of parotid cancer patients.

In summary, the present results demonstrate the occurrence of whole-body oxidative stress parotid gland tumor patients like in many other types of tumor [15–17]. However, the intensity of whole-body oxidative stress in tumor of the parotid gland is relatively low as only some markers of oxidative stress (protein thiols, AOPP, and FRAP) evidence its occurrence. Taking into account the organ affected, it seems that studies of oxidative markers in saliva could be more promising. Such a study is in progress.

## Figures and Tables

**Table 1:** Demographic characteristics of the examined groups.

Examined groups	Control (*n* = 24)	PA (*n* = 17)	WT (*n* = 9)	ACC (*n* = 8)
Age (years ± SD)	55.5 ± 9.4	53.6 ± 7.7	57.4 ± 6.8	61.5 ± 5.4
Female/male	16/8	12/5	5/4	6/2
Surgery type				
ECD	NA	1	0	0
Par I or II		3	4	0
Par I-II		9	1	1
Par I–III		2	2	0
Par I–IV		0	0	3
Mean tumor size (mm) (min–max)	NA	26 (13–55)	25 (12–40)	24 (18–30)
Tumor side, right/left	NA	11/4	4/3	2/2

PA: pleomorphic adenoma group; WT: Warthin's tumor group; ACC: acinic cell carcinoma group; ECD: extra-capsular dissection; Par: parotidectomy; NA: nonapplicable.

## Abbreviations

ABTS^*∗*^: 2,2′-Azinobis(3-ethylbenzothiazoline-6-sulfonic acid) free radical
ACC: Acinic cell carcinoma
AOPP: Advanced oxidation protein products
FRAP: Ferric Reducing Antioxidant Potential
MDA: Malondialdehyde
OS: Oxidative stress
PA: Pleomorphic adenoma
PBS: Phosphate-buffered saline
WT: Warthin's tumor.
